# Tissue and cellular tropism of *Eptesicus fuscus gammaherpesvirus* in big brown bats, potential role of pulmonary intravascular macrophages

**DOI:** 10.1177/03009858241244849

**Published:** 2024-04-15

**Authors:** Ursula G. Perdrizet, Janet E. Hill, LaRhonda Sobchishin, Baljit Singh, Champika Fernando, Trent K. Bollinger, Vikram Misra

**Affiliations:** 1 University of Saskatchewan, Saskatoon, SK, Canada

**Keywords:** bats, chiroptera, *Eptesicus fuscus*, gammaherpesvirus, *Patagivirus vespertilionid gammaherpesvirus 3*, pulmonary intravascular macrophage

## Abstract

Gammaherpesviruses (γHVs) are recognized as important pathogens in humans but their relationship with other animal hosts, especially wildlife species, is less well characterized. Our objectives were to examine natural *Eptesicus fuscus gammaherpesvirus* (EfHV) infections in their host, the big brown bat (*Eptesicus fuscus*), and determine whether infection is associated with disease. In tissue samples from 132 individual big brown bats, EfHV DNA was detected by polymerase chain reaction in 41 bats. Tissues from 59 of these cases, including 17 from bats with detectable EfHV genomes, were analyzed. An EfHV isolate was obtained from one of the cases, and electron micrographs and whole genome sequencing were used to confirm that this was a unique isolate of EfHV. Although several bats exhibited various lesions, we did not establish EfHV infection as a cause. Latent infection, defined as RNAScope probe binding to viral latency-associated nuclear antigen in the absence of viral envelope glycoprotein probe binding, was found within cells of the lymphoid tissues. These cells also had colocalization of the B-cell probe targeting *CD20* mRNA. Probe binding for both latency-associated nuclear antigen and a viral glycoprotein was observed in individual cells dispersed throughout the alveolar capillaries of the lung, which had characteristics of pulmonary intravascular macrophages. Cells with a similar distribution in bat lungs expressed major histocompatibility class II, a marker for antigen presenting cells, and the existence of pulmonary intravascular macrophages in bats was confirmed with transmission electron microscopy. The importance of this cell type in γHVs infections warrants further investigation.

Gammaherpesviruses (γHVs) have the ability to establish life-long infections in immune cells, and most host species examined are parasitized by one or more γHV.^15^ They are typically associated with disease when there is cross-species transmission or the host is immunosuppressed.^1,9^ There are 7 genera within this subfamily: *Rhadinovirus*, *Lymphocryptovirus*, *Percavirus*, *Patagivirus*, *Bossavirus*, *Manticavirus*, and *Macavirus*.^22,67^ Virus-host interaction for the human and mouse γHVs have been better characterized than those of other animals.^1^ For human and mouse members of Gammaherpesvirinae, infection is characterized by a 2-stage life cycle divided into latent and lytic, or productive, infection. In latency, the virus is found in a quiescent state where the genome is circularized, chromatinated, and tethered to the host DNA.^39^ During this latent state, only a few genes that promote and maintain latency are expressed. One gene which is particularly important in maintaining a latent state is the latency-associated nuclear antigen (LANA).^25^ Multiple triggers can reactivate the virus from this state into lytic replication where infectious virus is produced and released from cells.^58^ During lytic infection of cells by herpesviruses, viral gene expression occurs in an ordered manner and genes are categorized as immediate early, early, or late based on their temporal order of expression. The expression of late genes follows the initiation of viral genome replications, and these genes primarily encode proteins that make up the virion.^48^ The different gene expression profiles in latent and lytic infections can be used to identify the state of the virus within a host.

The genomes of several γHVs have recently been detected in bats.^11,32,44,49,52,66,70,72^ Isolation of these viruses from bats is rare (to date only 2 γHVs have been isolated from bat species) and little is known about their pathogenesis.^28,46,54,59^ Diseases associated with γHV infection in humans include lymphoproliferations, autoimmune disorders, and neoplasms.^20^ In bats, γHV infection has been associated with lesions but causation remains undetermined.^28,49^ *Eptesicus fuscus gammaherpesvirus* (EfHV, also called *Vespertilionid gammaherpesvirus 3*), the sole member of the *Patagivirus* genus,^59^ can be detected in the blood of 20% to 80% of captive and free ranging bats^23^ suggesting that there is a high prevalence of the virus in big brown bats. This virus can be propagated in cultured cell lines we have derived from big brown bats (*Eptesicus fuscus*).^2^

The cellular and tissue tropism of this virus are unknown. Sites of latency of other γHVs are primarily associated with B cells and sometimes macrophages or dendritic cells of lymphoid tissues in the spleen, thymus, and bone marrow.^68,71,75^ Although these are the major sites of latency, this viral state has also been reported in the lung of experimentally infected laboratory mice using murine herpesvirus 4.^30^ However, pulmonary intravascular macrophages (PIMs), which are found within the lung of other species, are absent in rodents.^69^ Pulmonary intravascular macrophages have been shown to support viral replication with other viruses, but a role for these cells in γHV infection has not been reported.^7,63^

Here, we characterize the expression of late viral genes under conditions thought to mimic lytic infections, describe the tissue and cellular distribution of EfHV genomes in naturally infected bats, and isolate and sequence the genome of a new isolate and variant of EfHV.

## Materials and Methods

### Case Description

Big brown bat cases were submitted for necropsy and routine diagnostic testing to the Western and Northern Regional Centre of the Canadian Wildlife Health Cooperative. From a total of 314 big brown bats submitted between 2017 and 2021, 130 cases had tissues suitable for polymerase chain reaction (PCR) screening for EfHV (Supplemental Table S1). Tissues were collected at the time of necropsy and frozen at −20°C until screening or fixed for histological evaluation. A new isolate of EfHV was obtained from one of these cases, a female big brown bat submitted for necropsy in 2020 by an animal rehabilitator for weakness of 1 month in duration.

### Polymerase Chain Reaction

One hundred and thirty cases were screened for γHV infection by PCR on DNA extracted (Qiagen DNeasy blood and tissue kit, cat. 69504) from pooled samples of liver, spleen, and lung. Not all tissues were well preserved; therefore, an internal control gene *GAPDH* was targeted in an initial PCR reaction to determine whether amplifiable DNA was present in the samples. For every PCR run, a no template control was included. Polymerase chain reaction was performed as previously described using primers targeting viral *BGLF4* and big brown bat *GAPDH*.^59^

### Histopathology

Hematoxylin and eosin-stained slides from 59 cases were reviewed; 17 of these were positive for EfHV viral DNA by PCR screening. These cases were chosen based on the availability of slides and good preservation of tissues. Tissues were fixed in 10% neutral-buffered formalin, embedded in paraffin, and stained with hematoxylin and eosin. After fixation, sections with bone were decalcified in 20% formic acid for less than 24 hours and then embedded in paraffin. The spleen and lymph node were assessed for lymphocyte hyperplasia, which was recorded as present or absent. Splenic lymphoid hyperplasia was defined as more than 50% of the white pulp containing secondary follicles.^42,61^ Hyperplasia of the lymph node could involve expansion of the paracortical region with lymphocytes or follicular hyperplasia with or without interfollicular proliferation.^35^

### Virus Isolation and Transmission Electron Microscopy

Virus isolation was performed from 1 case, a female bat with cytomegaly and karyomegaly of the tracheal epithelium. Pooled liver, lung, and kidney frozen at −20°C were homogenized in 1.5 mL of Dulbecco’s modified Eagle’s medium (Gibco, cat. 12430112) at 30 Hz for 4 minutes in a Retsch Mixer Mill with a 5.5 mm stainless steel grinding bead (MP Biomedicals, 116540431) and 0.1 g of silica beads (Fisher, cat. 360991112). The sample was centrifuged at 15 700 × *g* for 15 minutes. One milliliter of the supernatant was filtered using 0.2 μM polyethersulfone filter (Whatman, cat. 6780-2502) and added to a 75 cm^2^ flask (Sarstedt, cat. 83.3911.002) of passage 8 EfK3b cells in Dulbecco’s modified Eagle’s medium containing 10% fetal bovine serum, penicillin, streptomycin, and amphotericin B (Antibiotic-Antimycotic, Gibco, cat. 5240062). Cytopathic effect was first observed 4 days post-infection, and at 7 days post-infection, the flask had 95% cytopathic effect and was frozen at −80°C.

Virus was purified by serially centrifuging the supernatant at 4°C after 3 freeze-thaw cycles: 1500 × *g* for 5 minutes (Sorvall Legend RT, Thermo Scientific), 10 000 × *g* for 15 minutes (Sorvall RC6 Plus, Thermo Scientific, Waltham), and 80 000 × *g* for 1 hour (Sorvall Wx Ultra, Thermo Scientific). The pellet was resuspended in 50 μL of phosphate-buffered saline with 20 μL of 2% glutaraldehyde in 0.1 M sodium cacodylate stored at 4°C until imaging. Transmission electron microscopy (TEM) was performed as previously described with the Hitachi HT7700 transmission electron microscope.^59^

For cellular TEM, 3 flasks with 90% cytopathic effect were used. Media and trypsinized cells were centrifuged at 325 × *g* for 10 minutes (Sorvall legend, Thermo Scientific), and the pellet was resuspended in 36 mL phosphate-buffered saline and centrifugation was repeated. The pellet was fixed with 10 mL of 2% glutaraldehyde in 0.1 M sodium cacodylate and incubated at 4°C for 4 hours. Cells were pelleted with the previous centrifugation step and resuspended in 1 mL of 0.1 M sodium cacodylate. Measurements were taken with Image-Pro Premier version 9.3.3.

### Genome Sequencing

Virus was purified for sequencing as described earlier. Ultracentrifugation was performed with 6 mL of 30% w/v sucrose cushion and resuspended in 50 μL 10 mM Tris pH 8.5. This unique virus isolate EfHV/SK/02/2020 along with the isolate we use in our laboratory EfHV/SK/01/2016 were sequenced. The sequencing libraries were prepared using the Nextera XT Library Preparation Kit (Illumina, cat. FC-131-1024). The libraries were diluted to 8 pM and sequenced using a V2 500 cycle Nano Kit (Illumina, cat. MS-103-1003) on an MiSeq instrument. Reads were trimmed for quality using Trimmomatic^4^ sliding window 4:30 and minlen 36. Reads were assembled into contigs using SPAdes 3.12.0^47^ and mapped to the reference genome EfHV accession NC_040615 using Geneious Prime 2022.0.1. Gaps between contiguous sequences and several regions with multiple single nucleotide polymorphisms were confirmed using PCR with Top taq DNA polymerase (Qiagen, 200205), followed by purification of products using MinElute PCR purification kit (Qiagen, cat. 28004) or gel extraction and purification with the QIAquick gel extraction kit (Qiagen, cat. 28706), followed by Sanger sequencing (Macrogen). The sequencing data are available from GenBank accession number OM517184.

### In Vitro Lytic Gene Expression

To choose potential targets for determining tissue tropism of EfHV, viral gene expression in cell culture was quantified. Acyclovir was used to arrest cellular DNA synthesis to identify the late viral genes. EfK3b cells were maintained in Dulbecco’s modified Eagle’s medium (D-MEM, Gibco, cat. 12430112) supplemented with 10% fetal bovine serum (Corning, cat. 35-010-CV), 10 units/mL penicillin, and 10 μg/mL streptomycin (Gibco, cat. 15140122). They were infected with EfHV at a multiplicity of infection of 1 in the presence of 45 μg/mL acyclovir (Calbiochem, cat. 114798) for 1 hour at 37°C. Inoculum was removed and replaced with complete media containing the same concentration of acyclovir. Controls were treated in the same way without the addition of chemicals to the media. Cells were incubated at 37°C for 24 hours. Experiments were performed in triplicate and gene expression was normalized to a 4°C 1 hour infection control.

RNA was extracted using the QIAGEN RNeasy plus mini kit (cat. 74134), as per the manufacturer’s protocol. RNA (121 ng) from the acyclovir experiment was used for complementary DNA synthesis with the iScript gDNA clear cDNA synthesis kit (Bio-Rad, cat. 172-5034). The complementary DNA was diluted to a final volume of 200 μL, and 5 μL was used in the real-time PCR reaction with 15 μL of SsoFast EvaGreen supermix (Bio-Rad, cat 172-5204). Four micromoles of each primer were used for each of the viral open reading frames, as previously described.^59^ Briefly, the thermocycler program was enzyme activation at 95°C for 30 seconds, denaturation at 95°C for 5 seconds, annealing/extension at variable temperatures for 5 seconds, with the denaturation and annealing/extension repeated for 40 cycles. The melt curve was performed from 65 to 95°C in 2 second steps with 0.5°C increments.

### In Situ Hybridization

Probes were designed against viral nucleic acid sequences and can bind RNA or nonchromatinized DNA. These probes targeted the gene encoding the *envelope glycoprotein gp52* (V-Ef-gammaherpesvirus-gp52-C1 cat. 1070981-C1), which is expressed in productive infection, the gene encoding *LANA* (V-Ef-gammaherpesvirus-gp74-C2 cat. 1070971-C2), which is associated with latency, and the gene encoding the host B-cell protein *CD20* of big brown bats, *Eptesicus fuscus membrane spanning 4 domains A1* (Ef-MS4A1-C1 cat. 1070991-C1). Control probes included a positive control targeting the *peptidylprolyl isomerase B variant X1* transcript of big brown bats (Ef-PPIB-C1 cat. 1073191-C1) and a negative control *dihydrodipicolinate reductase* from bacteria (Negative control probe DapB cat. 310043). Cell culture control was used to test the specificity of the viral probes by infecting one of two 175 cm^2^ flasks of EfK3b cells at a multiplicity of infection of 1 and at 24 hours post-infection embedding cells in agarose prior to fixation in 10% neutral-buffered formalin. Both cell pellets were embedded in a single paraffin block. Serial sections of slides made from tissue blocks of submitted cases were probed in quadruplicate with the positive control *peptidylprolyl isomerase B*, negative control *dihydrodipicolinate reductase*, *gp52* and *LANA*, or *LANA* and *CD20*.

Slides from 9/41 PCR positive cases were chosen for in situ hybridization (ISH) based on the preservation of tissues, size of sections, and recentness of submission. The slide of each case containing the spleen was labeled in quadruplicate with positive control, negative control, *LANA* and *CD20*, and *gp52* and *LANA* probes. Additional tissues where viral probe binding was observed were occasionally present on the same slide (lymph node n = 2, bronchus-associated lymphoid tissue n = 4, and lung n = 7). One case was excluded because of weak labeling in the internal positive control for a total n = 8. Five micron tissue sections from formalin-fixed paraffin-embedded tissues were mounted on Superfrost Plus slides (Fisher Scientific cat. 12-550-15). They were prepared using the RNAScope 2.5 HD Duplex assay kit (ACDbio cat. 322430) following the manufacturer’s protocol with pretreatment in the target retrieval buffer for 15 minutes at 95 to 100°C and 15 minutes in protease plus treatment at 40°C for lymphoid tissues, lung sections, and cell culture. Two additional tissues of interest from slides that did not contain the spleen were also processed. This included the section of trachea with inclusion bodies from the bat from which EfHV/Saskatoon/02/2020 was isolated and a sagittal section of the head from the bat with *gp52* probe binding in the lung. For the sections of trachea and decalcified head, the pretreatment conditions were as follows: pretreatment in the target retrieval buffer for 15 and 30 minutes, respectively, at 95 to 100°C and 30 minutes protease plus treatment at 40°C.

Images were captured with an Olympus BX41 Infinity 5 camera and Infinity Analyze version 7.0.3.1111 software. To determine whether the probe binding for *LANA* was significant, the number of dots indicating probe binding within the lymphoid tissues were counted per 60× (field of view 0.37 mm^2^) for the slides probed with both *LANA* and *CD20* and the negative control.

### Immunohistochemistry

Immunohistochemistry (IHC) for major histocompatibility complex (MHC) class II alpha chain was performed by Prairie Diagnostic Services Inc using their commercially available HLA-DR-α chain clone TAL.1B5 (Agilent, cat no. M074601-2) on an automated staining platform (Autostainer Plus, Agilent Technologies Canada). Heat-induced epitope retrieval was performed, and the primary antibody was applied for 30 minutes at a 1:100 dilution. Antibody binding was detected using a horseradish peroxidase (HRP)-labeled polymer detection reagent (Envision+, Agilent Technologies Canada) and the labeling was visualized using 3.3’-diaminobenzidine tetrahydrochloride (Dako liquid DAB+ Substrate Chromogen System, Agilent Technologies Canada) as the chromogen. A positive control from diagnostic material was included.

### Transmission Electron Microscopy of Bat Lung

The hematoxylin and eosin–stained slide of lung with viral probe binding was submitted for TEM, as previously described.^3^ Briefly, the coverslip was removed, resin was applied to the area of interest on the slide, and 90 nm sections were made. The grids were stained with uranyl acetate and images were captured with the Hitachi HT7700 transmission electron microscope.

### Statistical Analysis

All the analyses were performed using Prism (v.9.3.1). Fisher’s exact test was used to determine whether there was an association between viral infection and lymphoid hyperplasia. The significance of probe binding in the lymphoid tissues was determined using a 2-tailed *t*-test without correction and cut-off of *P* < .05 comparing the average number of foci per field between the negative control and *LANA* or *gp52*. Multiple 2-tailed *t*-tests were used to compare gene expression at 24 hours post-infection in the presence or absence of acyclovir using the 2-stage step-up method and a false discovery rate of 1.00% and 4 degrees of freedom.

## Results

### Polymerase Chain Reaction and Histopathology

The purpose of examining cases of big brown bats presenting for necropsy was to determine whether there are any pathological changes associated with EfHV infection. Of the pooled tissues from 130 big brown bats submitted between 2017 and 2021, EfHV DNA was detected by PCR in 41 cases. Of those cases with good preservation, 59 had slides available for review, 17 of which were from bats infected with EfHV. All slides were assessed for neoplasia/tumors and proliferation of the lymphoid tissues. Splenic white pulp (n = 51) and lymph nodes (n = 27) were examined in these cases, and there was no association between EfHV infection and hyperplasia of the splenic white pulp or lymph nodes (*P* = .1023 and *P* > .999, respectively). The bronchus-associated lymphoid tissue was examined (n = 47), but no hyperplastic lesions were observed.

**Figure 1.**
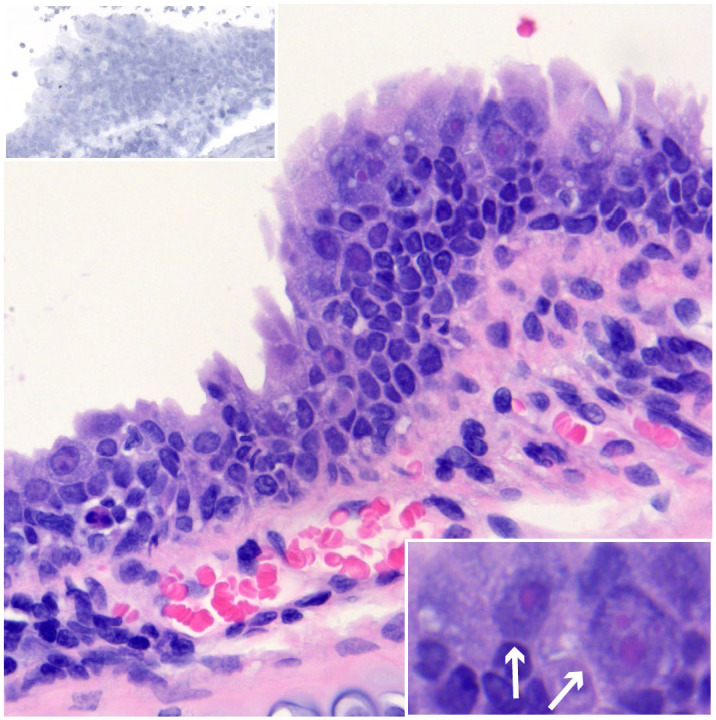
Epithelial dysplasia and intranuclear inclusion bodies, trachea, big brown bat. EfHV/SK/02/2020 was isolated from this bat. Case 126. Hematoxylin and Eosin (HE). Lower right inset: higher magnification demonstrating intranuclear inclusion bodies (arrows). HE. Upper left inset: serial section of the trachea negative for *Eptesicus fuscus gammaherpesvirus gp52* and *LANA. gp52* and *LANA* in situ hybridization.

Various neoplasms have been associated with γHV infections in other species, but no neoplasms were detected in this study. Two of the EfHV positive cases had large intranuclear inclusion bodies within the tracheal epithelium with features of dysplasia. From one of these cases, a unique isolate of EfHV/Saskatoon/02/2020 was cultured (Fig. 1). Since nuclear inclusion bodies are frequently associated with DNA viruses, we tested this case for adenovirus using IHC, which was negative.

### Virus Isolation and Genome Sequencing

In an attempt to isolate a virus from a big brown bat submitted to the Western Northern Regional Centre of the Canadian Wildlife Health Cooperative with large intranuclear inclusion bodies within the epithelium of the trachea, the supernatant of homogenized tissue was added to cultured EfK3b cells. The bat was female with interscapular fat stores, brittle long bones (osteopenia), and pale pectoral muscles. Histologically, there were basophilic or eosinophilic intranuclear inclusions within the tracheal epithelium accompanied by cellular and nuclear enlargement (cytomegaly and karyomegaly) and dysplasia.

Following the addition of the supernatant, the EfK3b cells became rounded, characteristic of herpesvirus cytopathic effect. Transmission electron microscopy and whole genome sequencing were used to identify the isolate. The virus displayed intranuclear replication in vitro and the negative stained virus was spherical, enveloped, and measured 105 ± 5 nm, which was consistent with it being a herpesvirus. A gammaherpesvirus (EfHV/SK/01/2016) had previously been isolated in our lab, so to confirm our isolate was unique and not a contaminant, we sequenced the genomes of both isolates for comparison. Illumina sequencing resulted in 464× coverage of the retained reads for EfHV/Saskatoon/02/2020 and 397× coverage for EfHV/Saskatoon/01/2016. The reference sequence NC_040615.1 used for comparison was created from the original virus isolate EfHV/SK/01/2016; this isolate had 99.9% nucleotide identity with the reference. The differences in the EfHV/SK/01/2016 nucleic acid sequence from the reference were characterized by 2 insertions, 1 transition, 2 transversions, 12 substitutions, and 2 insertions or deletions in tandem repeats. The nucleotide sequence identity of the isolate presented here (EfHV/SK/02/2020) was 99.7%. The variations in the nucleotide sequences between EfHV/SK/02/2020 and the reference included 3 insertions, 6 deletions, 61 transitions, 20 transversions, 10 substitutions, and 16 insertions or deletions in tandem repeats. The whole genome sequence for EfHV/SK/02/2020 was deposited in GenBank (accession number OM517184).

### In Vitro Lytic Gene Expression

To select gene targets that could aid in differentiating lytic infection from latency, we identified genes expressed at high levels late in the lytic cycle following the onset of viral DNA replication and whose expression was inhibited by blocking DNA replication. Infected cells were maintained in the absence or presence of acycloguanosine (acyclovir), which we had previously established inhibits EfHV DNA replication. The relative amounts of transcripts for various viral genes^59^ were then determined.

**Figure 2.**
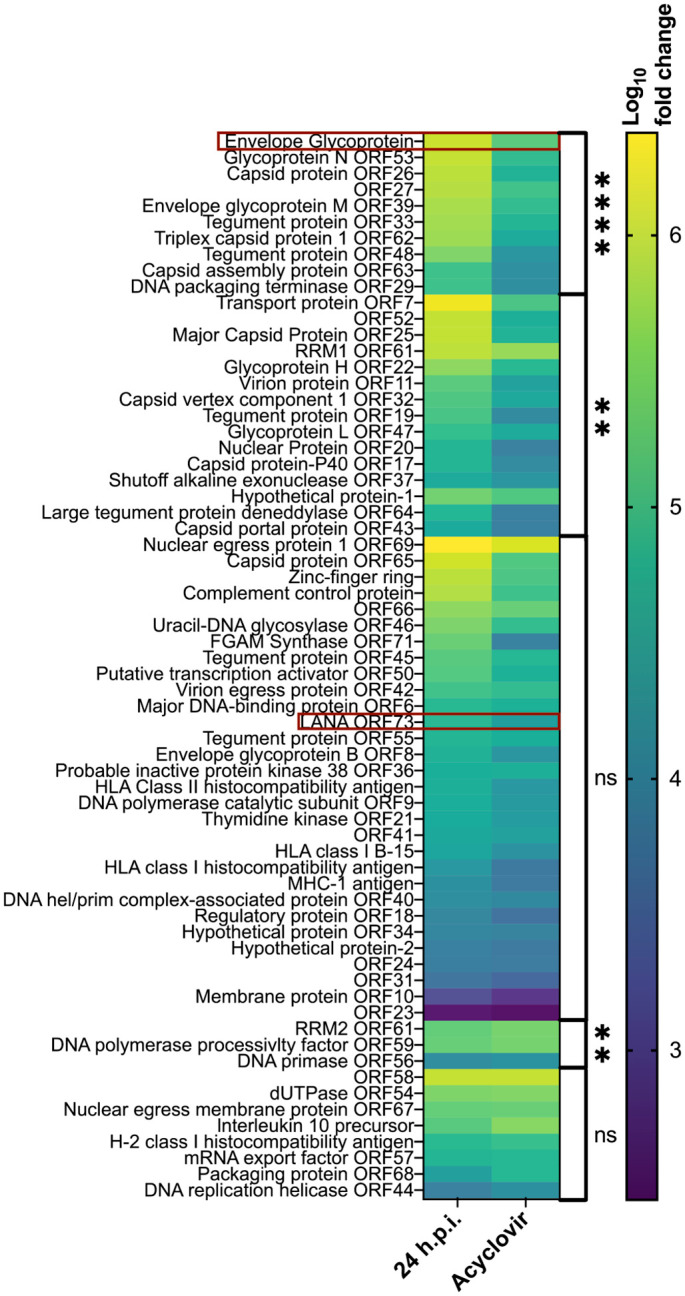
Heatmap of the log10 fold change of *Eptesicus fuscus gammaherpesvirus* (**EfHV)** genes relative to 4°C infection control. Gene expression was measured at 24 hours post-infection (h.p.i.) with or without acyclovir. They are ordered by gene expression for those that were inhibited by acyclovir at the top and those that were potentiated at the bottom, followed by a *P*-value comparing the 2 groups, then by fold change at 24 h.p.i. (highest to lowest). Red boxes indicate target genes for in situ hybridization probes. Statistical significance of the comparisons between the acyclovir treated and untreated cells at 24 h.p.i. are indicated to the right of the graph (ns, not significant; ****P < .0001; and **P < .01). RRM1, ribonucleoside-diphosphate reductase large subunit; RRM2, ribonucleoside-diphosphate reductase small subunit; LANA, latency-associated nuclear antigen.

Twenty-four hours after infection, all known genes were expressed (Fig. 2). Acyclovir treatment significantly decreased the expression of several genes. The gene with greatest expression and most inhibition with acyclovir treatment was *envelope glycoprotein gp52* (Fig. 2). We therefore selected *gp52* as the ISH probe target to identify cells undergoing a lytic infection.

### In Situ Hybridization and Immunohistochemistry

In situ hybridization was used to determine the localization of viral nucleic acids in tissues and the subtype of lymphocyte infected. The probes can bind complementary sequences in RNA and DNA but not chromatinized DNA. In latency, the genomes of yHVs are chromatinized; therefore, we would expect probe binding to represent viral RNA of the latent gene products. To detect latently infected cells, we probed for EfHV *LANA* nucleic acids; in other yHVs, this gene is required for the maintenance of latency. We expected latently infected cells to bind the *LANA* probe without binding the *gp52* probe. In productive infection the chromatinization of the genome is altered, and there is expression of lytic viral genes like our probe target *gp52* (Fig. 2). There is also the expression of latent genes like our latency probe *LANA*. With our methods we could not distinguish probe binding between viral RNA in productive infections and nonchromatinized viral DNA. Therefore, colocalization within a cell of both viral probes could indicate productive infection or nonchromatinized viral DNA. Excluding productive infections, nonchromatinized viral DNA would be found in cells during acute infections before chromatinization within the nucleus occurs or if virions or cells undergoing lytic viral replication were phagocytosed. The cellular probe targeted B lymphocytes (a site of latency for other γHVs) and was directed against the nucleic acids of the B-cell protein CD20. Although not entirely specific for B cells, *CD20* is expressed at high levels in lymphoid tissues.^16^ Latently infected B cells were defined as those in which probe colocalization of *CD20* and *LANA* occurred. Cells could not be probed with *gp52* and *CD20*, as this was a colorimetric assay capable of detecting 2 colors and these probes were assigned to the same color (Supplemental Figure S1).

**Figure 3.**
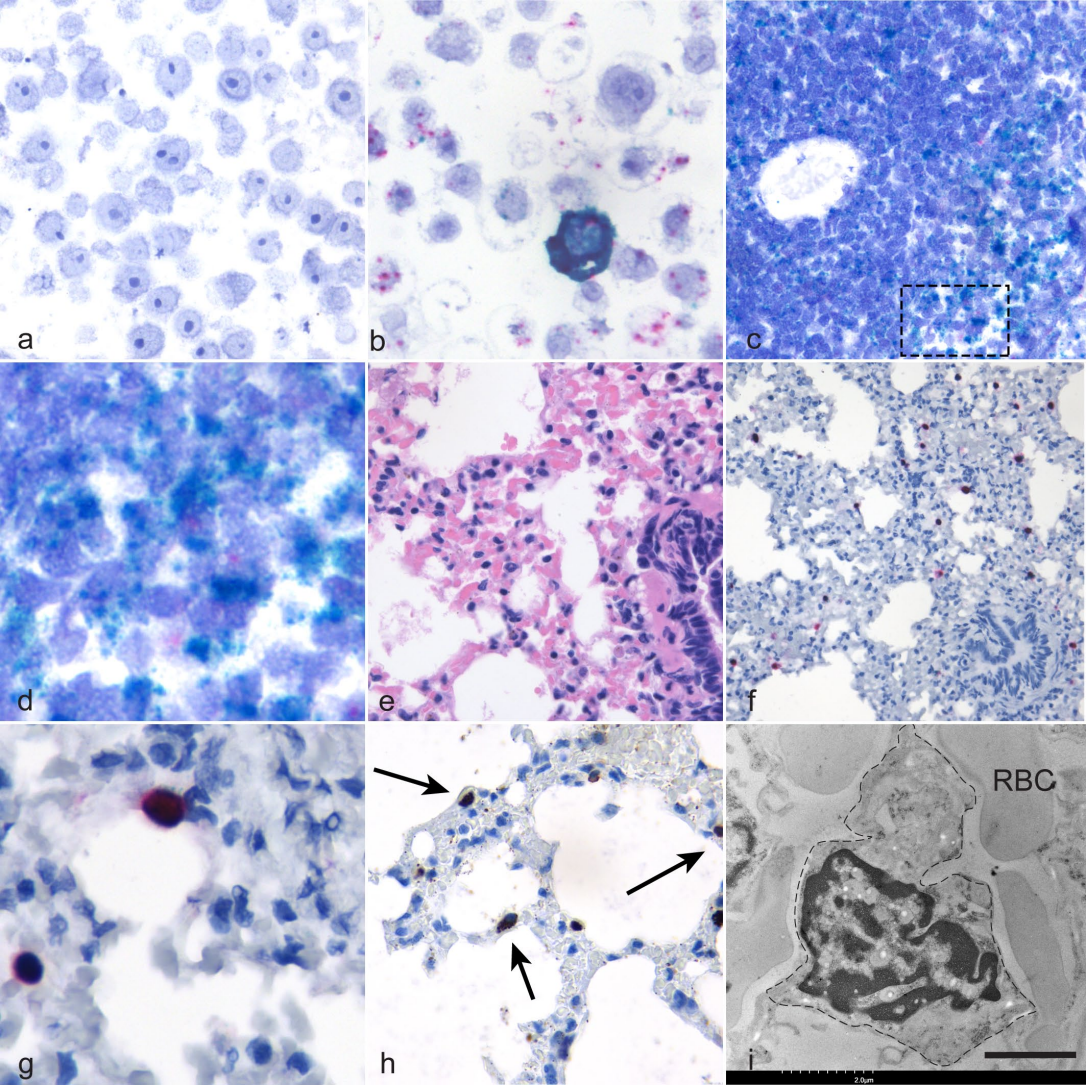
*Eptesicus fuscus gammaherpesvirus* (**EfHV) infection in cell culture and big brown bats.** (a, b) *Eptesicus fuscus* kidney cell line 3b (EfK3b) cells. *LANA* and *gp52* in situ hybridization (ISH). (a) No probe binding was observed in uninfected cells. (b) Colocalization of EfHV *LANA* (red) and *gp52* (teal) was observed in cells infected with EfHV. (c, d) Splenic white pulp from big brown bat 123. *LANA* and *CD20* ISH. (c) Colocalization of *LANA* (red) and *CD20* (teal) probes. (d) Higher magnification of the area surrounded by a dashed rectangle in (c). (e–i) Lung, big brown bat, case 129. (e) Hematoxylin and eosin. (f) Serial section demonstrating intense probe binding for *LANA* (red) and *gp52* (teal). *LANA* and *gp52* ISH. (g) Higher magnification showing colocalization of these probes in cells within alveolar capillaries. *LANA* and *gp52* ISH. (h) Serial section of lung in (e). Cells with major histocompatibility (MHC) class II immunolabeling (brown), as indicated by arrows, displayed similar pattern to the ISH in (f) and (g). MHC class II immunohistochemistry. (i) Electron photomicrograph of lung tissue from the same blocks as shown in (e–h) demonstrating a pulmonary intravascular macrophage outlined by a dashed line. RBC, red blood cell. Scale bar 2 µm.

To validate the viral probes, cell culture pellets from either uninfected cells or cells infected for 24 hours with EfHV were embedded in the same block and probed using *gp52* and *LANA*; positive and negative technical controls were similarly treated. There was minimal nonspecific probe binding in the uninfected cell pellet (Fig. 3a), whereas the infected cell pellet section bound *gp52* and *LANA* probes (Fig. 3b).

The probe binding for *LANA* in the spleen was significantly different from the negative control in 4 out of 8 cases with probe colocalization with the *CD20* probe in a subset of cells (Fig. 3c, d). No *gp52* labeling was observed in the spleens of these cases indicating there was no splenic viral reactivation. In bronchus-associated lymphoid tissues, all slides demonstrated probe binding to *LANA* and *CD20*, but only 2 of the 4 were significantly different from the negative controls. One of 2 lymph nodes evaluated had significantly greater amount of *LANA* probe binding than its complementary negative control.

Of the 7 lungs examined, one of the cases had strong probe binding of individual cells with both *gp52* and *LANA* (Fig. 3e–g). This was a male bat submitted in 2021 with ulcerative cellulitis and dermatitis. These cells were distributed uniformly within the alveolar capillaries, and they did not bind the *CD20* probe (Fig. 3f, g). To identify these cells, an antibody against MHC class II antigen was applied to a serial section of the lung. Epitope binding was observed in individual cells within the alveolar capillaries (Fig. 3h). A similar pattern of MHC class II antigen binding was observed in lung from an otherwise healthy EfHV PCR negative bat.

The intense *gp52* probe binding observed in the lung of 1 bat was interpreted as possible productive infection; therefore, the sagittal section of the head that included the nasal turbinates, salivary glands, and mucosa-associated lymphoid tissue was processed for ISH. Within the mucosa-associated lymphoid tissue, there was probe binding for *LANA* and *CD20*, but not for *gp52*.

### Transmission Electron Microscopy of Bat Lung

As PIMs were suspected as the cell type with intense gp52 probe binding, the hematoxylin and eosin–stained slide of the bat lung was submitted for TEM. Large cells with abundant cytoplasm and indented nuclei were adherent to the endothelial surface of alveolar capillaries, consistent with PIMs (Fig. 3i).^40^

## Discussion

Observing γHVs in their hosts is a challenge because most infections are latent and asymptomatic, making information about pathobiology with respect to establishment of latency, viral reactivation, and shedding scarce. Murine herpesvirus 4 infection in laboratory mice is the most commonly used model to study γHVs, but these mice are not a natural host and the virus is not easily transmitted to other mice.^17^ These challenges can be overcome by studying natural infections in other primary host species, like bats. We determined the tissue and cellular distribution of EfHV within naturally infected big brown bats. Latently infected cells were found within lymphocytes of lymphoid tissues, and individual cells in the alveolar capillaries of the lung are either involved in virus production or in the immune response to infection. No association could be established between viral infection and any pathological changes.

Using in vitro gene expression and combining this with what is known about other γHVs, we chose ISH probes that were able to determine cell and tissue distribution of EfHV nucleic acids with the potential to distinguish lytic and latent infections in wild bats. We regarded cells with binding of the EfHV *LANA* probe, a gene expressed during latency by related γHV, in the absence of probe binding for a viral structural glycoprotein *gp52*, as latently infected. The latent reservoir for EfHV was identified in lymphocytes of the lymphoid tissues expressing *CD20*. There was intense probe binding for *CD20* within germinal centers of the splenic white pulp indicating that they are most likely B lymphocytes.

The *gp52* probe localized within individual cells that were scattered throughout the alveolar capillaries, and these cells also bound the latent probe. No binding for the *CD20* probe was observed in these cells, but we cannot definitively rule out that these cells are not B lymphocytes. Some γHVs reactivate with differentiation of B cells into plasma cells which express variable amounts of the transcript for *CD20*.^45^ However, the distribution of these probe positive cells is most consistent with PIMs. The antibody used on sections of the lungs is a nonspecific marker for antigen presenting cells including macrophages, dendritic cells, monocytes, and B lymphocytes.^16^ Based on the distribution of epitope binding and location of these cells, PIMs were suspected as the cell type that bound the antibody.^40,53^ Similar antibody binding was found in the lungs of an uninfected big brown bat, suggesting that these cells are not exclusive to EfHV-infected big brown bats. As we could not differentiate non-chromatinized DNA or RNA with our methods, this probe binding could either represent productive viral infection or phagocytosis of infected cells or virions if these are PIMs. Taken together, the ISH results suggest that latency is present in B cells of lymphoid tissues (spleen, lymph node, bronchus-associated lymphoid tissues, and mucosa-associated lymphoid tissues), while cells of the lung are involved in infection, but it is unclear whether this represents productive infection, phagocytosis of virions, or virus-infected cells.

Supporting our hypothesis that these cells are PIMs we used TEM to demonstrate that cells with the morphological characteristics of PIMs are present in big brown bat lungs. Pulmonary intravascular macrophages have roles in several viral infections either supporting viral replication or in contributing to disease through their activation.^5–8,57^ Fibrosis of the lungs can be sequela of infection with several γHV infections like ovine herpesvirus 2 and equine herpesvirus 5, and lung changes have been associated with γHV infections in a broader range of species, but the role of these viruses in the pathogenesis of these lesions is uncertain.^13,27,29,41,65,73^ The role of PIMs in the pathobiology of these infections has not been studied, but there is evidence of macrophage involvement. In equine multinodular pulmonary fibrosis associated with equine herpesvirus 5 infections, alveolar macrophages contain viral inclusion bodies.^14,74^ Involvement of alveolar macrophages was not observed in our case.

Gammaherpesviruses in bats are common,^28,32,46,54^ but rarely are there any reports on the diseases associated with infection.^28,49^ The lack of pathological changes or association of lymphoid hyperplasia with γHV infection in our cases is not surprising because in infected but otherwise healthy individuals, these viruses are maintained in a latent state. Disease is usually only seen during acute infections or in latent infections with concurrent immunosuppression, and the typical lesion of lymphoid proliferation is a nonspecific finding with multiple causes. No conclusions could be made about the pathologic features of dysplasia with cytomegaly and karyomegaly of the tracheal epithelium and γHV infection given the small sample size, but we speculate that they are related to immunosuppression and a concurrent unidentified viral infection.

The unique isolate of EfHV/SK/02/2020 was distinct from the original isolate EfHV/SK/01/2016 used in our lab, as confirmed by whole genome sequencing. The differences in some of the nucleic acid sequences alter the predicted proteins of multiple open reading frames, but the significance of these differences requires further investigation. As viruses are not a homogeneous population and can mutate in cell culture, it is expected that after multiple passages there were several small differences in EfHV/SK/01/2016 to the sequence deposited in GenBank, only one of which altered the predicted amino acid sequence of an MHC class I antigen.

**Figure 4.**
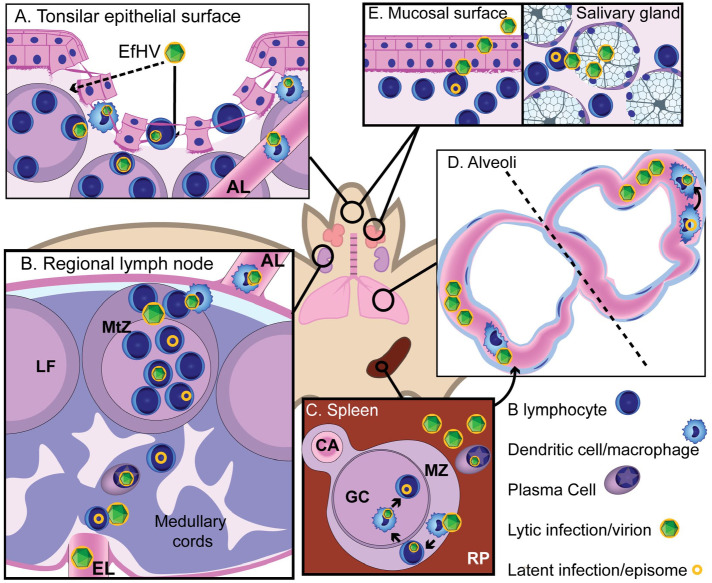
Proposed pathogenesis for *Eptesicus fuscus gammaherpesvirus* (EfHV). (A) New host contacts saliva containing virus primed for infecting B lymphocytes and dendritic cells (DC). Exposure to virions could occur through gaps in the epithelium of the tonsillar crypts (arrow) or transcytosis (dashed arrow). Infection of B lymphocytes and DCs occur. (B) DCs migrate to regional lymph node via the afferent lymphatics and infect follicular B cells and eventually memory B cells in the germinal center. Further systemic spread occurs by reactivation of latently infected B cells during differentiation into plasma cells. Dissemination is possibly by infected B cells or cell-free viremia. (C) The primary site of latency, the splenic white pulp, is colonized by infection of marginal zone macrophages then marginal zone B cells, follicular dendritic cells, and finally germinal center B cells through cell to cell spread. Differentiation of splenic B cells into plasma cells results in reactivation of gammaherpesvirus infection. (D) Pulmonary intravascular macrophages (PIMs) could be involved in the viral cycle in a number of ways: phagocytosing virus or virus-infected cells following splenic reactivation, becoming infected during phagocytosis, or being directly involved in productive infection, either via latently infected migratory monocytes, which then differentiate into PIMs, or resident PIMs that reactivate virus. (E) Virus is shed from epithelial or glandular cells that are infected by B cells. B cells are infected by migrating macrophages or cell-free virus produced from a myeloid cell, or they reactivate virus following latent infection. Abbreviations: AL, afferent lymphatic; CA, central arteriole; EfHV, *Eptesicus fuscus gammaherpesvirus*; EL, efferent lymphatic; GC, germinal center; LF, lymphoid follicle; MtZ, mantle zone; MZ, marginal zone; RP, red pulp.

Based on our findings, we propose a model for the infectious cycle of EfHV in *E. fuscus*, its primary host (Fig. 4). This is largely conjectural and is based on our observations as well as the literature on other γHVs.

### Transmission

Our previous work indicates that EfHV is shed from the oral cavity of juvenile bats (unpublished data) suggesting that the virus may be transmitted in saliva. For γHVs, there are multiple potential modes of transmission but those that are applicable to wild bats would include contact with saliva, inhalation, or sexual transmission.^43,68^ Epstein-Barr virus is shed in saliva and primed for infecting B cells.^33,55^ Gaps within the epithelium and basement membrane of tonsillar crypts would allow direct viral access to susceptible cells^34,50^ or virus might reach susceptible cells via transcytosis through epithelial cells.^64^ We excluded the olfactory epithelium as the point of entry since natural transmission via this route has not been demonstrated.^19,24^

### Colonization

Based on the literature for murine herpesvirus 4, spread from the primary site of infection to regional lymph nodes occurs through serial myeloid to lymphoid transmission.^21^ How γHVs go on to colonize the spleen is less certain and could possibly occur through cell-free viremia, but more likely occurs through cell-associated transfer.^12,18,36–38,68^ Splenic colonization occurs through cell to cell spread from the marginal zones to germinal centers.^18^

### Reactivation

The final steps of reactivation and viral shedding are not as well characterized. Experimentally, reactivation occurs at the primary site of infection.^31^ Reactivation at the most likely primary sites of infection was not observed in our case. Big brown bats maintain latent infections in B cells of the lymphoid tissues, but no reactivation was detected here. We only demonstrated the possibility of lytic infection in what are likely PIMs, but the lung is only reported as a primary site of infection under experimental conditions.^30,60^ Pulmonary intravascular macrophage probe binding could represent phagocytosis of virus or infected cells following reactivation at a different site, possibly leading to infection and virus replication in PIMs; synchronous reactivation from latency in PIMs infected in situ or in monocytes that then migrate and differentiate into PIMs; or acute infection at the dissemination phase. The first explanation is favored because no latently infected macrophages were identified in the lung in other cases, the spleen did not have hybridization consistent with latent infection, and we speculate that reactivation occurred here followed by infection of the PIMs. This bat had significant comorbidities and was likely immunocompromised favoring reactivation. The role of these cells in γHV infection would be overlooked solely studying rodents and humans because they have not been reported in the former and occur at low numbers in the latter.^40,69^

### Shedding

As no viral lytic probe binding was observed in the oral cavity, the following is based on peer-reviewed literature. Virus is transferred from lymphocytes to the epithelium.^56,62^ In vivo viral shedding has been demonstrated from salivary glands^51^ and not from the oral mucosa,^26^ but either or both sites are possible. We favor reactivation at secondary sites either directly in PIMs or with PIMs as an intermediate step resulting in the production of virus that could reinfect lymphocytes in mucosa-associated lymphoid tissues maintaining the serial lymphoid-myeloid transmission. Cells in the mucosa-associated lymphoid tissues would then in turn infect epithelial cells.

There were many limitations to our study. The cell culture experiments need to be interpreted with care as γHV gene expression is not always conserved between cell lines, within a cell culture, and in the host. In addition, RNA expression was measured and protein expression should be used to support the findings. We were restricted to bats submitted to the Western and Northern Regional Centre of the Canadian Wildlife Health Cooperative with no possibility of experimental infection. As they were natural infections, the timing of infection relative to sampling is unknown. We could not control for the effects of concurrent illnesses in lymphoid tissues possibly obscuring an association with lymphoid hyperplasia. The variations between samples and individual characteristics of the spleen led to an inconsistent number of fields examined by ISH of latent infection between cases. With ISH we cannot differentiate between viral nucleic acids in the cytoplasm or in endolysosomes of macrophages. Although pretreating our slides with DNase would discriminate between the RNA and DNA of EfHV, we would not be able to rule out the possibility of the RNA being from a phagocytosed cell. However, porcine PIMs are more cytolytic and have limited phagocytic capability relative to alveolar macrophages.^10^ As our results are observational, we cannot conclude with certainty on the significance of our findings to the pathogenesis of the γHV.

Future directions for research include investigating the role of PIMs in γHV dissemination, transmission, lung disease, and immunity; examining the pathogenesis of γHVs following viral reactivation; and examining how primary or secondary sites contribute to viral transmission. Gammaherpesviruses can also be used as a proxy measure for stress and to understand the role between stress and viral shedding. To study virus-host interactions in bats, and how stress contributes to viral transmission using this γHV, development of bat cell lines (B lymphocytes and PIMs) that supports latent infection is crucial.

## Supplemental Material

sj-pdf-1-vet-10.1177_03009858241244849 – Supplemental material for Tissue and cellular tropism of Eptesicus fuscus gammaherpesvirus in big brown bats, potential role of pulmonary intravascular macrophages Supplemental material, sj-pdf-1-vet-10.1177_03009858241244849 for Tissue and cellular tropism of Eptesicus fuscus gammaherpesvirus in big brown bats, potential role of pulmonary intravascular macrophages by Ursula G. Perdrizet, Janet E. Hill, LaRhonda Sobchishin, Baljit Singh, Champika Fernando, Trent K. Bollinger and Vikram Misra in Veterinary Pathology
