# Supplementary material for: Tissue and cellular tropism of Eptesicus fuscus gammaherpesvirus in big brown bats, potential role of pulmonary intravascular macrophages
Source: Vet Pathol. 2024 Apr 15;61(4):550–61. doi: 10.1177/03009858241244849 (PMC11264566; doi:10.1177/03009858241244849)
Supplement: sj-pdf-1-vet-10.1177_03009858241244849 – Supplemental material for Tissue and cellular tropism of Eptesicus fuscus gammaherpesvirus in big brown bats, potential role of pulmonary intravascular macrophages [file sj-pdf-1-vet-10.1177_03009858241244849.pdf]

Supplemental Materials
Tissue and cellular tropism of Eptesicus fuscus gammaherpesvirus in big brown bats, potential role of pulmonary intravascular macrophages

Ursula G Perdrizet, Janet E Hill, LaRhonda Sobchishin, Baljit Singh, Champika Fernando, Trent K Bollinger, Vikram Misra

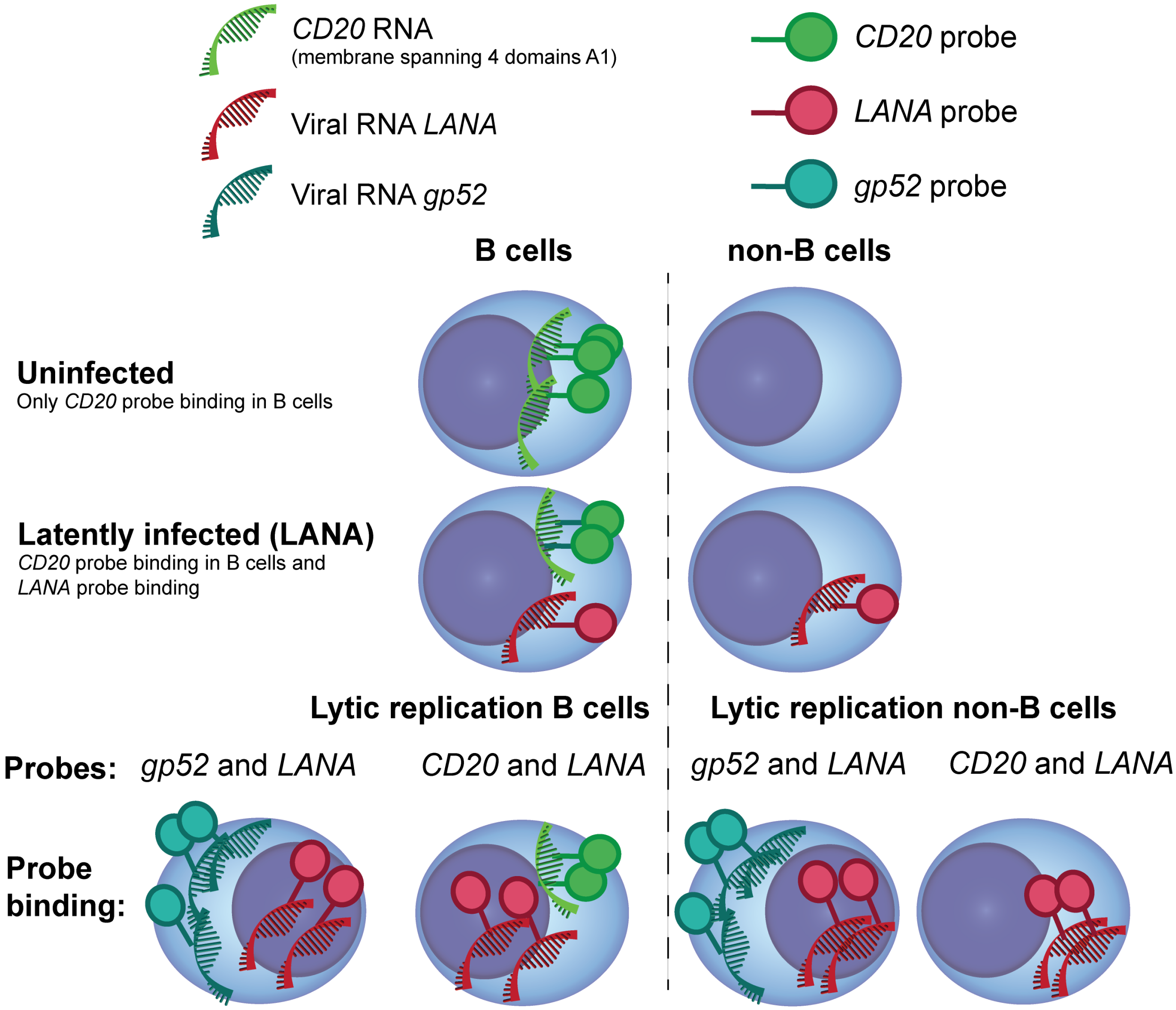

Supplemental Figure S1. Schematic representation of in situ hybridization (ISH) results. The colorimetric ISH assay only has two colors available to identify probes. Probes were used in combination to identify either latent infection in B cells (*LANA* and *CD20*) or lytic replication (*gp52* and *LANA*). The *CD20* and *gp52* probes developed the same color, so serial sections were used to determine if similar *CD20* when *gp52* probe binding distributions were identified.

Supplemental Table S1: Big brown bat cases submitted to the Canadian Wildlife Health Copperative Western Northern Regional Centre from Saskatchewan, Canada between 2017 and 2021 and

EfHV PCR Result	ID	Age	Sex	Weight (g)	Date Found	Death Date	Date Received	Captivity Category (days)	Storage	Mortality State	City	Diagnosis
Negative	1	Juvenile	Male	12.1	2017-01-12	2017-01-14	2017-01-16	Rehab(2)	Frozen	Found Live, Died	Saskatoon	N/A
	2	Adult	Male	6.9	2017-01-14	2017-04-01	2017-04-04	Rehab(88)	Frozen	Found Live, Died	North Battleford	Emaciation
	3	Adult	Female	16	2017-04-12	2017-04-12	2017-04-12	Free-ranging	Fresh	Euthanized	Saskatoon	Rabies
	4	Adult	Female	17.3	2016-12-05	2017-04-13	2017-04-13	Rehab(129)	Fresh	Live	Saskatoon	Frost bite
	5	Adult	Male	13.5	2017-04-03	2017-04-08	2017-04-20	Free-ranging	Frozen	Found Live, Died	Saskatoon	N/A
	6	Adult	Female	16.4	2017-04-19	2017-04-19	2017-04-20	Free-ranging	Fresh	Found Dead	Saskatoon	N/A
	7	Adult	Female	18.5	2016-10-04	2017-04-01	2017-04-20	Rehab(179)	Frozen	Found Dead	Saskatoon	N/A
	8	N/A	Male	12.2	N/A	2017-05-13	2017-05-13	Free-ranging	N/A	Found Dead	Saskatoon	N/A
	9	Adult	Male	16.2	2017-04-16	2017-06-20	2017-06-20	Rehab(65)	Fresh	Euthanized	Grasswood	N/A
	10	Adult	Male	11.2	2017-06-26	2017-06-26	2017-06-27	Free-ranging	Fresh	Euthanized	Saskatoon	Trauma
	11	Adult	Male	15.2	2017-05-30	2017-07-07	2017-07-07	Rehab(38)	Fresh	Live	Saskatoon	N/A
	12	N/A	Male	15.6	2017-07-11	2017-07-11	2017-07-11	Free-ranging	Fresh	Live	Saskatoon	Misadventure
	13	N/A	N/A	9.3	2017-07-23	2017-07-24	2017-07-24	Free-ranging	Fresh	Live	Hague	Misadventure
	14	Adult	Male	16.1	2017-08-07	2017-08-07	2017-08-15	Free-ranging	Fresh	Euthanized	Saskatoon	Trauma
	15	N/A	Male	18.8	2017-08-10	2017-08-10	2017-08-11	Free-ranging	Fresh	Found Dead	Saskatoon	Predation
	16	Juvenile	Male	15.3	2017-08-08	2017-08-12	2017-08-14	Rehab(4)	Frozen	Found Live, Died	Saskatoon	Rabies
	17	Juvenile	Male	13	2017-08-19	2017-08-20	2017-08-22	Free-ranging	Fresh	Euthanized	Spiritwood	Trauma
	18	Juvenile	Male	13.9	2017-08-24	2017-08-24	2017-08-25	Free-ranging	Fresh	Found Dead	Saskatoon	Trauma
	19	Juvenile	Male	9.6	2017-08-24	2017-08-25	2017-08-25	Rehab(1)	Fresh	Live	Aberdeen	Rabies
	20	Adult	Male	15.5	2017-08-23	2017-08-24	2017-08-25	Rehab(1)	Fresh	Found Live, Died	Saskatoon	Rabies, trapped
	21	Juvenile	Female	15.2	2017-08-23	2017-08-24	2017-08-25	Rehab(1)	Fresh	Found Live, Died	Saskatoon	Trapped
	22	N/A	Female	N/A	2017-08-30	2017-08-30	2017-08-31	Rehab(1)	Frozen	Found Live, Died	Saskatoon	Predation
	23	Juvenile	Female	12.6	2017-08-23	2017-09-01	2017-09-01	Free-ranging	Fresh	Live	Saskatoon	Trapped
	24	N/A	N/A	N/A	2017-09-25	2017-09-25	2017-09-25	Free-ranging	Fresh	Found Live, Died	Clavet	Trauma
	25	Adult	N/A	17.3	2017-10-01	2017-10-01	2017-10-02	Free-ranging	Fresh	Found Dead	Saskatoon	Rabies
	26	Adult	Female	26.6	2017-11-19	2017-11-19	2017-11-21	Free-ranging	Frozen	Found Live, Died	Saskatoon	Predation
	27	Adult	Female	N/A	N/A	2017-12-12	2017-12-13	Free-ranging	Fresh	Euthanized	Saskatoon	Misadventure
	28	N/A	Female	16.1	N/A	2018-02-23	2018-02-23	Free-ranging	Frozen	Found Dead	Saskatoon	Cause of death unknown
	29	Adult	Male	12	2018-03-11	2018-03-11	2018-03-12	Free-ranging	Fresh	Found Dead	Saskatoon	Emaciation
	30	N/A	Female	10.7	2017-12-13	2018-03-14	2018-04-03	Rehab(91)	Frozen	Found Live, Died	Prince Albert	Bacterial pneumonia
	31	N/A	Female	15.1	2018-02-12	2018-02-14	2018-04-03	Rehab(2)	Frozen	Found Live, Died	Saskatoon	Cause of death unknown
	32	Adult	Male	11.1	2018-05-01	2018-05-02	2018-05-03	Free-ranging	Fresh	Euthanized	Saskatoon	Trauma
	33	Adult	Female	16.1	2018-05-06	2018-05-07	2018-05-07	Free-ranging	Fresh	Euthanized	Saskatoon	Trauma
	34	N/A	Male	N/A	2018-05-15	2018-05-15	2018-05-16	Free-ranging	N/A	Found Live, Died	Saskatoon	Cause of death unknown
	35	N/A	Female	12.2	2018-05-14	2018-05-15	2018-05-16	Clinic(1)	Fresh	Found Live, Died	Saskatoon	Sepsis
	36	N/A	Male	11.5	2018-05-15	2018-05-16	2018-05-16	Free-ranging	Fresh	Euthanized	Saskatoon	Rabies, predation
	37	Adult	Male	11.6	2018-06-09	2018-06-09	2018-06-11	Free-ranging	Fresh	Euthanized	Saskatoon	Rabies, predation
	38	Adult	Male	11.4	2018-06-15	2018-06-15	2018-06-15	Free-ranging	Fresh	Euthanized	Waldheim	Misadventure
	39	Adult	Male	14.8	2018-06-22	N/A	2018-06-22	Free-ranging	Frozen	Found Dead	Saskatoon	Predation
	40	Adult	Male	17	2018-07-08	2018-07-08	2018-07-09	Free-ranging	Frozen	Euthanized	Saskatoon	Predation
	41	N/A	Male	20.4	2018-07-07	2018-07-07	2018-07-09	Free-ranging	N/A	Found Live, Died	Furdale	Predation
	42	Adult	Male	20	2018-07-15	2018-07-15	2018-07-18	Free-ranging	Frozen	Euthanized	Saskatoon	Predation
	43	Adult	Male	13	2018-08-02	2018-08-02	2018-08-02	Free-ranging	Fresh	Euthanized	Saskatoon	Trauma
	44	Juvenile	Male	11	2018-07-24	2018-07-25	2018-08-03	Rehab(1)	Frozen	Found Live, Died	Saskatoon	Rabies
	45	Adult	Male	13	2018-07-27	2018-07-27	2018-08-03	Rehab(1)	Frozen	Found Live, Died	Saskatoon	Predation
	46	Adult	Male	13	2018-07-22	2018-08-03	2018-08-03	Rehab(12)	Fresh	Live	Saskatoon	Trapped
	47	Adult	Male	18	2018-08-05	2018-08-05	2018-08-07	Free-ranging	Frozen	Euthanized	Leask	Predation
	48	Adult	Male	22	2018-08-08	2018-08-08	2018-08-15	Free-ranging	Frozen	Found Dead	Warman	Drowning
	49	Adult	Male	16	2018-08-15	N/A	2018-08-16	Free-ranging	Fresh	Live	Saskatoon	Misadventure
	50	Juvenile	Male	11	2018-08-14	2018-08-14	2018-08-16	Free-ranging	N/A	Found Dead	Saskatoon	Predation
	51	N/A	N/A	N/A	2018-08-22	2018-08-23	2018-08-23	Free-ranging	Fresh	Euthanized	Saskatoon	Trauma
	52	Adult	Male	17	N/A	2018-08-31	2018-08-31	Free-ranging	Fresh	Live	Wakaw	Misadventure
	53	N/A	N/A	N/A	N/A	2018-09-04	2018-09-06	Free-ranging	N/A	Found Live, Died	Delisle	Predation
	54	Juvenile	Female	12	N/A	2018-10-05	2018-10-05	Rehab	N/A	Found Live, Died	Shields	Rabies
	55	Adult	Male	14	N/A	2018-10-20	2018-10-22	Free-ranging	N/A	Euthanized	Saskatoon	Predation
	56	Adult	Male	N/A	N/A	2018-11-07	2018-11-08	Free-ranging	N/A	Euthanized	Saskatoon	N/A
	57	Adult	Male	17	N/A	2018-11-26	2018-11-27	Free-ranging	N/A	Euthanized	Saskatoon	Frost bite
	58	Juvenile	Male	15	N/A	2018-11-28	2018-11-28	Free-ranging	N/A	Found Dead	Prince Albert	Trauma
	59	Juvenile	Female	17	N/A	2018-12-05	2018-12-11	Free-ranging	N/A	Found Live, Died	Saskatoon	Hypothermia
	60	Adult	Female	16	2018-12-16	2018-12-16	2018-12-17	Free-ranging	N/A	Found Live, Died	Saskatoon	Misadventure
	61	N/A	Male	17	2019-01-14	2019-01-15	2019-01-15	Rehab(1)	N/A	Found Dead	Saskatoon	Misadventure
	62	Adult	Male	14	2019-01-30	2019-02-01	2019-02-01	Rehab(3)	N/A	Found Live, Died	Saskatoon	Misadventure
	63	N/A	Female	17	2019-02-14	2019-02-22	2019-02-21	Free-ranging	N/A	Found Dead	Saskatoon	Rabies
	64	Juvenile	Male	8	2018-10-19	2019-01-31	2019-02-21	Rehab(103)	N/A	Found Live, Died	Saskatoon	Cause of death unknown
	65	Adult	Male	15	2019-04-16	2019-04-17	2019-04-17	Free-ranging	Fresh	Live	Saskatoon	Trauma
	66	Adult	Male	12	2019-04-21	2019-04-22	2019-04-22	Free-ranging	N/A	N/A	Saskatoon	Trauma
	67	Adult	Male	N/A	2019-02-28	2019-05-13	2019-05-13	Rehab(74)	Fresh	Live	Saskatoon	Eptesipox virus
	68	Adult	Female	15	2019-05-15	2019-05-16	2019-05-15	Free-ranging	Fresh	Live	Saskatoon	Chronic suppurative arthritis of right elbow
	69	Adult	Female	20	2019-02-25	2019-05-27	2019-05-25	Rehab(91)	N/A	Euthanized	Saskatoon	Trauma
	70	Adult	Female	13	2019-05-28	2019-05-29	2019-05-29	Free-ranging	N/A	Found Live, Died	saskatoon	Cause of death unknown
	71	Adult	Male	10	2019-08-06	2019-08-06	2019-08-06	Free-ranging	Fresh	Live	Saskatoon	N/A
	72	N/A	Male	16	2019-09-09	2019-09-11	2019-09-10	Free-ranging	N/A	Euthanized	Melville	Misadventure
	73	Adult	Female	N/A	2020-01-15	2020-01-15	2020-01-15	Free-ranging	N/A	Euthanized	Saskatoon	Bacterial cellulitis and myositis with emaciation
	74	N/A	Male	12	2019-12-20	2020-03-13	2020-03-13	Rehab(84)	N/A	Found Dead	Martensville	Cause of death unknown

	75	Adult	Female	16	2020-04-15	2020-04-15	2020-04-15	Free-ranging	N/A	Euthanized	Saskatoon	Predation
	76	Adult	Female	15	2020-04-25	2020-04-27	2020-04-27	Rehab(2)	N/A	N/A	Saskatoon	Trauma
	77	Adult	Female	14	2020-04-28	2020-04-29	2020-04-29	Free-ranging	N/A	N/A	Saskatoon	Misadventure
	78	Adult	Male	13	N/A	2020-07-04	2020-07-04	Free-ranging	N/A	Euthanized	Saskatoon	Trauma
	79	Adult	Male	11	2020-07-06	2020-07-06	2020-07-07	Free-ranging	N/A	Euthanized	Saskatoon	Fibrinosuppurative arthritis right carpus
	80	Adult	Male	12	2020-06-28	2020-07-08	2020-07-08	Rehab(30)	N/A	Live	Saskatoon	Eptesipox virus
	81	Adult	Female	22	2020-07-29	2020-07-29	2020-07-30	Free-ranging	N/A	Euthanized	Saskatoon	Predation
	82	Adult	Female	20	2020-08-20	2020-08-20	2020-08-20	Free-ranging	N/A	Euthanized	Saskatoon	Misadventure
	83	N/A	Female	24.5	2020-10-19	2020-10-19	2020-10-19	Free-ranging	Fresh	Live	Rosthern	Misadventure
	84	Adult	Female	21.1	2020-11-03	2020-11-03	2020-11-04	Free-ranging	N/A	Euthanized	Saskatoon	Trauma
	85	Juvenile	Male	4	2020-10-22	2020-11-19	2020-11-19	Rehab(28)	Fresh	Dead	Saskatoon	Cause of death unknown
	86	Adult	Male	12.9	2020-11-23	2020-12-09	2020-12-09	Rehab(16)	Fresh	Live	Saskatoon	Trauma
	87	N/A	Female	14.9	2020-11-10	2021-01-14	2021-01-14	Rehab(65)	Fresh	Live	Saskatoon	Trauma
	88[d]	N/A	Female	14.7	2020-11-10	2021-01-14	2021-01-14	Rehab(65)	Fresh	Live	Saskatoon	Trauma
	89	Adult	Male	15.2	2020-10-31	2021-01-28	2021-01-28	Rehab(89)	N/A	Found Dead	Saskatoon	Trauma
	Unlabelled	N/A	N/A	N/A	N/A	N/A	N/A	N/A	N/A	N/A	N/A	N/A
	90	Adult	Male	12	2017-03-16	2017-03-17	2017-03-17	Rehab(1)	Fresh	Live	Saskatoon	Misadventrue
	91	Adult	Male	10	2017-03-28	2017-03-29	2017-03-29	Rehab(1)	Fresh	Live	Saskatoon	Trauma
	92	Adult	Female	10	2017-03-11	2017-03-25	2017-03-29	Rehab(14)	Frozen	Found Live, Died	Saskatoon	Trauma
	93	Adult	Male	9	2017-03-20	2017-04-03	2017-04-04	Rehab(14)	Frozen	Found Live, Died	Saskatoon	Emaciation
	94	Adult	Male	N/A	2017-04-07	2017-04-07	2017-04-07	Free-ranging	Fresh	Live	Saskatoon	Trauma
	95	Adult	Male	14.8	2017-04-08	2017-04-13	2017-04-13	Rehab(5)	Fresh	Live	Saskatoon	Frost bite
	96	Adult	Male	14.6	2017-04-05	2017-04-20	2017-04-20	Rehab(15)	Fresh	Live	Saskatoon	Trauma
	97	Adult	Male	13.9	2017-04-20	N/A	2017-04-20	Free-ranging	Fresh	Found Dead	Saskatoon	Cause of death unknown
	98	Adult	Male	9.9	2017-05-02	2017-05-03	2017-05-02	Rehab(1)	Fresh	Live	Holbein	Frost bite
	99	N/A	Male	11.3	N/A	2017-05-13	2017-05-15	Free-ranging	Fozen	Euthanized	Saskatoon	N/A
	100	Adult	Female	9.9	N/A	2017-05-20	2017-05-24	Free-ranging	Fresh	Found Dead	Saskatoon	N/A
	101	N/A	N/A	N/A	N/A	N/A	2017-07-07	Rehab	Frozen	Found Live, Died	Saskatoon	N/A
	102	N/A	N/A	N/A	N/A	N/A	2017-07-07	Rehab	Frozen	Found Live, Died	Saskatoon	N/A
	103	N/A	N/A	N/A	N/A	N/A	2017-07-07	Rehab	Frozen	Found Live, Died	Saskatoon	N/A
	104	N/A	N/A	N/A	N/A	N/A	2017-07-07	Rehab	Frozen	Found Live, Died	Saskatoon	N/A
	105	Adult	Female	24	2017-07-07	2017-07-07	2017-07-10	Free-ranging	Fresh	Euthanized	Saskatoon	Predation
	106	Juvenile	Female	16.4	2017-08-13	2017-08-13	2017-08-14	Free-ranging	Fresh	Euthanized	Saskatoon	Misadventure
	107	Adult	Male	17.7	2017-08-23	2017-08-23	2017-08-24	Free-ranging	Fresh	Euthanized	Saskatoon	Trapped
	108	N/A	N/A	N/A	2017-08-24	2017-08-24	2017-08-25	Free-ranging	Fresh	Found Dead	Saskatoon	Cause of death unknown
	109	Juvenile	Male	12.5	2017-08-24	2017-08-24	2017-08-25	Free-ranging	Fresh	Found Dead	Saskatoon	Cause of death unknown
	110	Juvenile	Male	12.5	2017-08-23	2017-08-24	2017-08-25	Rehab(1)	Fresh	Found Live, Died	Saskatoon	Trapped
	111	Juvenile	Female	16.4	2017-08-29	2017-08-29	2017-08-30	Free-ranging	Fresh	Euthanized	Saskatoon	Trauma
Positive	112	Juvenile	Male	11	2017-09-03	2017-09-05	2017-09-05	Free-ranging	Fresh	Live	Landis	Predation
	113	Juvenile	Male	14	2017-09-06	2017-09-06	2017-09-07	Free-ranging	Fresh	Live	Saskatoon	Predation
	114	Juvenile	Female	16	2017-09-20	N/A	2017-09-22	Rehab(2)	N/A	Euthanized	Saskatoon	Rabies
	115[a]	Adult	Male	15.7	2017-10-16	2017-10-16	2017-10-16	Free-ranging	Fresh	Euthanized	Saskatoon	Misadventure
	116	N/A	Female	9.2	2018-01-09	2018-03-14	2018-04-03	Rehab(64)	Frozen	Found Live, Died	Saskatoon	Emaciation
	117	N/A	Female	12.2	N/A	2018-05-11	2018-05-14	Free-ranging	Fresh	Found Live, Died	Saskatoon	Misadventure
	118	Adult	Female	12	2018-06-09	2018-06-09	2018-06-11	Free-ranging	N/A	Found Live, Died	Saskatoon	Rabies
	119[a]	Adult	Male	18.2	2018-06-11	2018-06-11	2018-06-11	Free-ranging	Fresh	Unknown	Saskatoon	Trauma
	120	Adult	Male	14	2018-07-10	2018-07-12	2018-07-24	Rehab(2)	Frozen	Found Live, Died	Saskatoon	Rabies
	121	Adult	Male	16	2018-07-30	2018-07-30	2018-07-30	Free-ranging	Frozen	Euthanized	Saskatoon	Rabies
	122	Adult	Male	20	2018-08-02	2018-08-02	2018-08-02	Free-ranging	Frozen	Found Dead	Saskatoon	Trauma
	123[a]	Juvenile	Male	13	2018-08-06	2018-08-07	2018-08-07	Captive (1)	Fresh	Live	Saskatoon	Misadventure
	124	Adult	Female	13	2019-04-19	2019-04-19	2019-04-22	Free-ranging	N/A	Euthanized	Vonda	Ulcerative stomatitis and tracheitis
	125[a]	Adult	Female	23.1	2019-11-04	2019-11-04	2019-11-04	Free-ranging	N/A	Euthanized	Saskatoon	Misadventure
	126[ab]	Adult	Female	15	2020-04-09	2020-05-04	2020-05-04	Rehab(25)	N/A	Live	Saskatoon	Osteopenia, tracheal dysplasia with cyto and karyomegaly
	127[a]	Juvenile	Male	12.7	2020-09-16	2020-09-29	2020-09-29	Rehab(13)	N/A	Live	Dalmeny	Rabies
	128[a]	Juvenile	Male	16.6	2020-10-25	2020-10-26	2020-10-26	Rehab(1)	N/A	Live	Saskatoon	Misadventure
	129[acd]	Adult	Male	14.6	2021-01-07	2021-01-18	2021-01-18	Rehab(11)	N/A	Found Live, Died	Saskatoon	Ulcerative celullitis and dermatitis

[a] EfHV *in situ* hybridization

[b] EfHV/SK/02/2020 isolated from liver, lung, and spleen homogenate from this bat

[c] EfHV lytic probe binding in lung

[d] MHC II IHC

N/A not available
